# Y‐box binding protein 1 in small extracellular vesicles reduces mesenchymal stem cell differentiation to osteoblasts—implications for acute myeloid leukaemia

**DOI:** 10.1002/jev2.12417

**Published:** 2024-03-18

**Authors:** Venkatesh Kumar Chetty, Jamal Ghanam, Kristína Lichá, Alexandra Brenzel, Dirk Reinhardt, Basant Kumar Thakur

**Affiliations:** ^1^ Department of Pediatrics III University Hospital Essen Essen Germany; ^2^ Institute of Molecular Biomedicine, Faculty of Medicine Comenius University in Bratislava Bratislava Slovakia; ^3^ Imaging Center Essen (IMCES) University Hospital Essen Essen Germany; ^4^ European Liquid Biopsy Society Hamburg Germany

**Keywords:** acute myeloid leukaemia, bone marrow microenvironment, mesenchymal stem cells, osteoblasts, small extracellular vesicles, Y‐box binding protein 1

## Abstract

Small extracellular vesicles (sEVs) released by acute myeloid leukaemia (AML) cells have been reported to influence the trilineage differentiation of bone marrow‐derived mesenchymal stem cells (BM‐MSCs). However, it remains elusive which biological cargo from AML‐sEVs is responsible for this effect. In this study, sEVs were isolated from cell‐conditioned media and blood plasma using size‐exclusion chromatography and ultrafiltration and characterized according to MISEV2018 guidelines. Our results demonstrated that AML‐sEVs increased the proliferation of BM‐MSCs. Conversely, key proteins that are important for normal haematopoiesis were downregulated in BM‐MSCs. Additionally, we revealed that AML‐sEVs significantly reduced the differentiation of BM‐MSCs to osteoblasts without affecting adipogenic or chondrogenic differentiation. Next, LC‐MS/MS proteomics elucidated that various proteins, including Y‐box‐binding protein 1 (YBX1), were upregulated in both AML‐sEVs and BM‐MSCs treated with AML‐sEVs. Clinically relevant, we found that YBX1 is considerably upregulated in most paediatric AML patient‐derived sEVs compared to healthy controls. Interestingly, sEVs isolated after the downregulation of YBX1 in AML cells remarkably rescued the osteoblastic differentiation of BM‐MSCs. Altogether, our data demonstrate for the first time that YBX1 containing AML‐sEVs is one of the key players that disrupt the normal function of bone marrow microenvironment by reducing the osteogenic differentiation of BM‐MSCs.

## 1 INTRODUCTION

Small extracellular vesicles (sEVs) are 30–200 nm lipid bilayer‐enclosed nanovesicles containing unique biological cargo and are released by all cell types across the extracellular space (Chetty et al., 2022; Ghanam et al., 2022). sEVs are involved in cell‐cell communication in various disease contexts by transferring their cargoes from one cell to another (Chetty et al., 2022; Lotvall & Valadi, 2007; Torralba et al., 2018). Especially in the tumour microenvironment, cancer‐derived sEVs play a pivotal role by acting as a signal mediator between tumour and stromal cells to promote cancer progression (Liu et al., 2006), angiogenesis (Nazarenko et al., 2010; Umezu et al., 2014) and metastasis (Hoshino et al., 2015).

Acute myeloid leukaemia (AML) is the most aggressive leukaemia characterized by chromosomal rearrangements and gene mutations in which bone marrow makes a multitude of abnormal blood cells called AML blasts or myeloblasts (Huang et al., 2020; Rubnitz et al., 2010). For years, it has been known that there is a crosstalk between AML blasts and critical components in the bone marrow microenvironment (BMM), resulting in abnormal proliferation and differentiation blockage of stem cells, which disrupts the normal haematopoiesis leading to bone marrow failure and eventually death, if left untreated (Goulard et al., 2018; Sendker et al., 2021; Yao et al., 2021). BMM is a complex niche that contains various cell populations, including endothelial cells, hematopoietic stem cells (HSCs), mesenchymal stem cells (MSCs), fibroblasts, osteoblasts, adipocytes and many immune cells (Duarte et al., 2018; Hughes et al., 2022).

Around 15% to 20% of all paediatric acute leukaemia cases correspond to only paediatric AML, in which many patients develop chemoresistance during remission, resulting in relapse (de Rooij et al., 2015; Lagunas‐Rangel et al., 2017). Among various components in the BMM, bone marrow‐derived MSCs (BM‐MSCs) serve as a critical essential factor since the reciprocal interaction between AML cells and BM‐MSCs is crucial for AML progression and contributes to treatment failure or success (Hanahan & Coussens, 2012; Jacamo et al., 2014). Besides the involvement of other extracellular factors, recent studies demonstrated that sEVs such as exosomes released by AML cells induced molecular changes in BM‐MSCs that suppressed the residual haematopoietic function in the BMM leading to poor prognosis and chemoresistance (Chen et al., 2019; Huan et al., 2013; Kumar et al., 2018; Zhang et al., 2022). Although these studies showed that AML‐sEVs influence the trilineage differentiation of BM‐MSCs in BMM (Kumar et al., 2018; Zhang et al., 2022), they have not adequately demonstrated which sEV cargo derived from AML‐sEVs is responsible for this effect. Thus, we addressed this phenomenon extensively in the current study.

Since sEVs are complex with various cargoes, including proteins, nucleic acids and lipids, it is challenging to unveil the cargo derived from AML‐sEVs that influences the normal function of BMM. Nevertheless, we recently demonstrated exclusively that EV‐chromatin derived from AML‐sEVs causes p53 dysfunction in BM‐MSCs through the overexpression of the p53‐negative regulator MDM2 (Ghanam et al., 2023).

In the current study, we identified that Y‐box binding protein 1 (YBX1) is considerably upregulated in most paediatric AML patient‐derived sEVs compared to healthy controls, and their expression level is independent of AML subtype. YBX1 is a multifunctional protein that is reported to be implicated in tumour progression and metastasis (El‐Naggar et al., 2015; Kwon et al., 2018; Somasekharan et al., 2015). Supporting this fact, we found that YBX1 containing sEVs derived from AML reduces the osteoblastic differentiation of BM‐MSCs. Altogether, our study opens new avenues in understanding the mechanism of AML‐derived sEVs in altering the homeostasis of BMM. Further research focusing on the functional interaction of AML‐derived sEVs with other components in BMM is crucial to developing a new therapeutic strategy for AML.

## 2 MATERIALS AND METHODS

### 2.1 Cell culture

Acute myeloid leukaemia (AML) and chronic myeloid leukaemia (CML) cell lines such as MV4‐11 and K562 were cultured in RPMI 1640 media (Gibco, Paisley, UK) containing 10% fetal bovine serum (FBS) and 1% Penicillin/Streptomycin (P/S) in a humidified incubator at 37°C and 5% CO_2_. Healthy bone marrow‐derived mesenchymal stem cells (BM‐MSCs) developed from a healthy male of 15 years old were obtained from AML‐BFM laboratory, Universitätsklinikum Essen. Healthy BM‐MSCs were cultured in DMEM high glucose media (Gibco, Paisley, UK) containing 20% FBS and 1% P/S under the same culture conditions.

For small extracellular vesicles (sEVs) isolation, MV4‐11, and K562 cells were seeded in 8 × 145 cm^2^ dishes containing 25 mL of RPMI 1640 media, each with 10% EV depleted FBS (FBS18) and 1% P/S and cultured for 72 h under the same culture conditions. FBS18 was obtained by centrifuging FBS at 100,000 × *g* for 18 h in Ultracentrifuge to deplete serum‐associated EVs. Following 72 h, cell conditioned media (CCM) was centrifuged at 500 × *g* for 10 min and 3000 × *g* for 20 min (4°C) to remove cell debris and apoptotic bodies. Cell lines utilized in this study were authenticated using STR fingerprinting service from IDEXX BioAnalytics, Germany, and mycoplasma contamination check was performed routinely using PCR.

### 2.2 Isolation and characterization of sEVs

**FIGURE 1:**
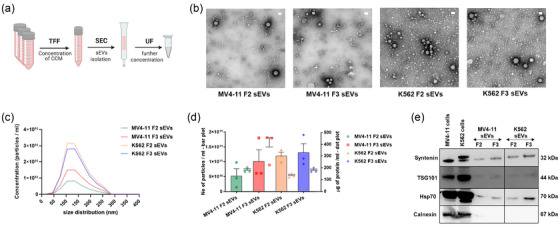
Isolation and characterization of leukaemia sEVs. (a) Steps involved in the sEVs isolation using tangential flow filtration, size‐exclusion chromatography and ultrafiltration, collectively named as TSU. (b) TEM imaging of sEVs derived from MV4‐11 and K562 cell lines. Scale bars‐ 100 nm. (c) Graph showing the particle size distribution of MV4‐11 and K562 sEVs determined by NTA. (d) Mean values of particle count evaluated using NTA (left y‐axis, bar plot), and free protein concentration determined using micro‐BCA (right y‐axis, dot plot). Data correspond to the mean ± S.E.M. obtained from three independent experiments. (e) Detection of canonical EV markers (syntenin, TSG101 and Hsp70) and EV negative marker (calnexin) using Western blot. The black outer line box implies the samples that were run together.

As previously described (Chetty et al., 2022), sEVs were isolated from 0.2 µm filtered MV4‐11 and K562 CCM using the combination of Tangential Flow Filtration (TFF‐ Easy; Hansa Biomed, Tallin, Estonia), Size Exclusion Chromatography (SEC; qEV10 column; IZON Science, Christchurch, NZ) and Ultrafiltration (Amicon Ultra‐4 10 kDa centrifugal filter, Merck Millipore, Darmstadt, Germany), collectively known as TSU (Figure 1a). After analysing various fractions obtained from CCM using TSU, we have previously determined that sEVs were enriched more in fractions 2 and 3 (F2 and F3) compared to other fractions, with less lipoprotein contamination. The individual yield of F2 and F3 can vary, however, the size of both F2 and F3 falls under the size range of sEVs and they have similar cargo profiles. Therefore, we included only F2 and F3, no other fractions, for the characterization (Chetty et al., 2022).

As previously described by Chetty et al. (2022), MV4‐11 and K562 sEVs (F2 and F3) were characterized by nanoparticle tracking analyser (NTA), micro‐BCA assay, transmission electron microscopy (TEM) and western blot. NTA and micro‐BCA were employed to determine the particle number per millilitre and the free protein concentration in sEV preparation, respectively. Following negative staining of sEVs with phosphotungstic acid, they were visualized in TEM at the Electron Microscopy Unit (EMU) of the Imaging Centre Essen (IMCES). TEM 16‐bit images were acquired using EMMENU image software (Version 4.09.83). Western blot (WB) was carried out with sEVs and whole‐cell lysate executing the same semidry procedure and staining with EV canonical markers (syntenin, TSG101 and Hsp70) and EV negative marker (calnexin).

As explained by Ghanam et al. (2023), sEVs were also isolated from fifteen peripheral blood plasma samples of paediatric AML patients and paediatric healthy control donors using a combination of Size Exclusion Chromatography and Ultrafiltration. AML patient samples (P1‐P15) were collected in the AML‐BFM laboratory at the University Hospital Essen in 2022, and their details are mentioned in Table 1. Paediatric healthy control donor samples (C1‐C15) were obtained from the Department of Paediatrics III, University Hospital Essen. Oral and written consent was obtained from all the paediatric AML patients and healthy donors prior to sample collection following the protocol approved by the ethics committee of the Medical Faculty, University Hospital Essen (16‐7069‐BO).

**TABLE 1:** Information about the paediatric AML patients involved in our study.

Sample ID	Patient ID	Age at sample collection	AML subtype	Phase	Point of treatment
P1	pAML‐166	15	M4	Initial	Before treatment
P2	pAML‐8	18	M4	Initial	Before treatment
P3	pAML‐243	2	M4	Initial	Before treatment
P4	pAML‐229	11	M4	Initial	Before treatment
P5	pAML‐59	12	M4	Initial	Before treatment
P6	pAML‐126	17	M4	Initial	Before treatment
P7	pAML‐298	12	M1	Initial	Before treatment
P8	pAML‐10	14	M1	Initial	Before treatment
P9	pAML‐236	11	M1	Initial	Before treatment
P10	pAML‐233	11	M1	Initial	Before treatment
P11	pAML‐145	18	M5	Initial	Before treatment
P12	pAML‐238	17	M5	Initial	Before treatment
P13	pAML‐26	3	M5	Initial	Before treatment
P14	pAML‐23	16	M5	Initial	Before treatment
P15	pAML‐259	1	M5	Initial	Before treatment

We have already optimized and know that we obtain more sEVs from blood plasma using our isolation method in fractions (2‐4) (Ghanam et al., 2023). Therefore, we mixed these fractions equally and used them for subsequent experiments.

### 2.3 Labeling of sEVs with PKH26 and subsequent transfer into BM‐MSCs

PKH26 Red Fluorescent Cell Linker Mini Kit for General Cell Membrane (Sigma Aldrich, Germany) was utilized to label MV4‐11 and K562 sEVs. 1 mM of PKH26 dye was prepared using Diluent C provided in the kit. Thirty microliters of isolated sEV fractions were treated with 5 µM of PKH26 dye in Diluent C and incubated at room temperature for 4 min. Then, the labelling reaction was stopped by adding 1% BSA in DPBS. Unlabelled PKH26 dye was removed by loading the samples into IZON qEV original column (IZON Science, Christchurch, NZ). For microscopy and flow cytometry analysis, fractions (F2 and F3) containing PKH26 labelled sEVs were mixed and added to BM‐MSCs at an approximately 50:1 ratio (sEVs/recipient cells). Only DPBS in place of sEVs was used as a negative control. After 24 h, the cells were fixed and permeabilized, and afterward, the nuclear and cell membrane staining were executed using DAPI (Abcam, Cambridge, UK) and WGA conjugated with Alexa 488 (Invitrogen, Germany), respectively. The next day, cells were visualized in confocal microscopy and analysed using Image J. On the other hand, PKH26‐labelled sEVs transferred into BM‐MSCs were analysed by flow cytometry.

### 2.4 Cell viability and proliferation assays

To evaluate if leukaemia sEVs influence the viability and proliferation of BM‐MSCs, different concentrations of MV4‐11 and K562 sEVs were added to BM‐MSCs. After 24 h, cell viability was checked using 3‐(4,5‐ dimethylthiazol‐2‐yl)‐2,5‐diphenyltetrazolium bromide (MTT) assay by measuring absorbance at 570 nm using a Tecan Infinite 200 Microplate Photometer (Tecan, Switzerland). In addition, apoptosis assay was performed using BD Pharmingen™ FITC Annexin V Apoptosis Detection Kit (BD Biosciences, Heidelberg, Germany) following the kit protocol to determine the percentage of apoptotic or necrotic cells due to the sEVs treatment.

To measure the proliferation potential of BM‐MSCs treated with leukaemia sEVs, colony‐forming unit assay was performed. 100 BM‐MSCs were seeded in StemMACS™ MSC Expansion Media, human (Miltenyi Biotech, Germany) with or without leukaemia sEVs. After 3 weeks of plating, colony numbers were counted following 0.5% crystal violet staining (Sigma Aldrich, Germany).

### 2.5 Quantitative real‐time PCR

BM‐MSCs treated with leukaemia sEVs were screened for various gene targets that are important for the normal hematopoietic supporting function of BM‐MSCs. Total RNA was extracted from untreated and treated BM‐MSCs using Qiagen RNA mini kit (Qiagen, Germany). Five hundred nanograms of RNA were subjected to reverse transcription using Transcriptor First Strand cDNA Synthesis Kit (Roche, Germany). Quantitative real‐time PCR was carried out in StepOnePlus™ Real‐Time PCR System (Applied Biosystems, USA) using FastStart Universal SYBR Green Master mix (Roche, Germany) and 200 nM primers specific for reference gene (GAPDH) or other target genes at 95°C for 10 min, then 95°C for 15 s, and 60°C for 1 min × 40 cycles. Sequences of forward and reverse primers are listed in Supplementary Excel Sheet 1.

After data acquisition, CT value of GADPH was used for the normalization. First, the ΔCT value was calculated for each sample by subtracting the average CT value of the target gene from the average CT value of GAPDH. Then, the mRNA level of each target relative to GAPDH was calculated using 2^ΔCT^. All the measurements were performed in triplicates (experimental replicates) and the same experiment was repeated for at least three times.

### 2.6 MSC trilineage differentiation analysis

BM‐MSCs treated with or without leukaemia sEVs were analysed for trilineage differentiation by flow cytometry using antibodies such as mouse anti‐human FABP4 (Cat No: ab93945, dilution‐1:100, Abcam, Cambridge, UK) + goat anti‐mouse IgG H&L with Alexa488 (Cat No: ab150113, dilution‐ 1:2000, Abcam, Cambridge, UK); rabbit anti‐human aggrecan Alexa647‐conjugated polyclonal antibody (Cat No: NB100‐74350AF647, dilution: 1:100, Novus Biologicals, CO, USA) and mouse anti‐human osteocalcin PE‐conjugated monoclonal antibody (Cat No: IC1419P, dilution‐ 1:50, Biotechne, Germany). Human MSC Functional Identification Kit (Cat No: SC006, Biotechne, Germany) was utilized to perform MSC trilineage differentiation analysis using confocal microscopy. Manufacturer protocol was exactly followed, and the images were captured after 21 days of culture.

### 2.7 LC‐MS/MS proteomics and data analysis

LC‐MS/MS proteomics was performed at the EMBL Proteomics Core Facility (Heidelberg, Germany) for the leukaemia sEVs alone and BM‐MSCs treated with leukaemia sEVs. Please refer to our previous publication (Ghanam et al.,2023) for the complete steps involved in sample preparation, LC‐MS/MS, data processing and data analysis.

### 2.8 Evaluation of YBX1 concentration using ELISA

YBX1 concentration was measured on sEVs derived from various cell lines, including leukaemia, and on BM‐MSCs treated with leukaemia sEVs. Human YB1 SimpleStep ELISA Kit (#ab269544, Abcam, Cambridge, United Kingdom) was employed for this application.

### 2.9 Proteinase K digestion

To study whether YBX1 is located inside or on the surface of the vesicles, AML and non‐AML derived sEVs were incubated with and without 600 mAU/mL Proteinase K (Thermofisher Scientific, Massachusetts, USA) for 1 h at 37°C with gentle shaking for every 15 min. Then, the reaction was stopped using the appropriate concentration of EDTA‐free protease inhibitor cocktail (Roche, Germany) by incubating the samples at RT for 10 min. Afterward, sEVs incubated with Proteinase K samples were subsequently treated with 0.1% Triton X (Sigma Aldrich, Germany) to lyse the membrane to quantify the level of YBX1 protein located inside the vesicles using ELISA.

### 2.10 Downregulation of YBX1

Neon™ Transfection System 100 µL Kit (Invitrogen, Germany) was used to perform the electroporation‐based transfection of MV4‐11 cells. Before electroporation, MV4‐11 cells were cultivated in RPMI media until they reached 70%–80% confluence. On the day of transfection, 5 million cells resuspended in 100 µL of Opti‐MEM media along with 10 µL of either si‐YBX1 (Cat No: 4390824, Thermofisher Scientific, Germany) or si‐scramble (Cat No: sc‐37007, Santa Cruz Biotechnology, Germany) (2 µM‐ final concentration) were electroporated under various pulse conditions (1200 V+, 1350 V+, and 1500 V + 35 ms + 1 pulse) using Neon™ Transfection System (Invitrogen, Germany). After electroporation, the transfected cells were seeded in a 60 mm Petri dish containing 10 mL of RPMI media. To assess the transfection efficiency, plasmid‐encoding GFP was utilized. Suitable pulse condition to downregulate YBX1 expression in MV4‐11 cells was determined using ELISA, western blot and qRT‐PCR. In addition, cell viability was also assessed by using MTT assay (Sigma Aldrich, Germany) and absorbance was measured at 540 nm. Once the right pulse condition was determined, the same experiment was repeated in more Petri dishes to isolate sEVs from MV4‐11 si‐YBX1 and MV4‐11 si‐scramble cells.

### 2.11 Alizarin red staining

For osteogenic differentiation, BM‐MSCs were seeded at 4200 cells/cm^2^ in a standard 24‐well plate and let grow until they reached 50%−70% confluency. Afterward, osteogenic differentiation media was added every 3–4 days for 21 days to induce osteogenesis. After 21 days, cells were washed twice with PBS, fixed with ice‐cold 70% ethanol for 1 h, and stained with 1 mL of 40 mM Alizarin red (Sigma Aldrich, Germany) for 10 min at RT. Images were acquired using Axio Observer Z.1 in transmitted light with the AxioCam 506.

### 2.12 Generation of pDESTmyc empty vector

pDESTmyc empty vector was generated from pDESTmycYBX1 by digesting it with SmaI and EcoRV (New England Biolabs, Germany). SmaI and EcoRV restriction digestion would enable the removal of YBX1 insert from pDESTmycYBX1. pDESTmycYBX1 was a gift from Thomas Tuschl (Addgene plasmid # 19878; http://n2t.net/addgene:19878; RRID:Addgene_19878) (Landthaler et al., 2008). Please refer to Figure S1 for the complete plasmid maps.

Total 2 µg of pDESTmycYBX1 was digested with 10 U of SmaI and EcoRV and incubated at 37°C for 1 h. Then, the sizes of both digested and undigested plasmid DNA were confirmed using agarose gel electrophoresis. Afterward, the digested DNA fragment of pDESTmycYBX1 without YBX1 insert containing SmaI and EcoRV (5966 bp) was excised from the agarose gel using a clean scalpel. DNA was extracted from the agarose gel using FastGene Gel/PCR extraction kit (Nippon Genetics, Germany) following the manufacturer's protocol, and DNA concentration was measured using NanoDrop 2000C (Thermofisher Scientific, Germany).

Around 75 ng of digested DNA fragment was then ligated using Quick Ligase Kit (New England Biolabs, Germany) following the kit protocol. Ligated DNA was subsequently transformed into one‐shot TOP10 chemically competent *E. coli* cells. The transformation mixture was spread onto an LB agar plate containing ampicillin. The next day, a single colony was picked up, and DNA was extracted from this colony using FastFilter plasmid DNA mini kit (Omega Bio‐Tek, USA) following the manufacturer's standard protocol. Extracted DNA was sent for DNA sequencing (Microsynth Seqlab, Göttingen, Germany) to confirm the removal of YBX1 insert containing SmaI and EcoRV sites.

### 2.13 Overexpression of YBX1 in BM‐MSCs

BM‐MSCs seeded in 12well plate were transiently transfected with 2.5 µg of pDESTmycYBX1 or pDESTmyc empty vector using 5 µL of P3000 reagent and 1.5 µL of Lipopectamine 3000 reagent following manufacturer's protocol (Invitrogen, Germany). For the transfection control, mock transfection was performed only with transfection reagents. After 48 h, transfected BM‐MSCs were collected, and YBX1 expression was analysed by western blot. In addition, confocal microscopy‐based osteogenic differentiation analysis was performed on the above‐mentioned transfected BM‐MSCs after 21 days of culture using human MSC Functional Identification Kit (Biotechne, Germany) following manufacturer's protocol.

### 2.14 Statistical analysis and reproducibility

GraphPad Prism 10 (GraphPad Software, San Diego, California, USA) was utilized to perform the statistical analyses using unpaired two‐tailed t‐test or one‐way ANOVA, Brown‐Forsythe test, and Bartlett's test. Dunnett's multiple comparisons test was executed to assess the statistical significance of various samples in reference to healthy control sEVs. *p*‐value less than 0.05 was considered statistically significant.

### 2.15 Data presentation

All the flowcharts presented in this study were created with BioRender.com. In addition, all the other figures were aligned or positioned in place using the same website.

## 3 RESULTS

### 3.1 Characterization of leukaemia sEVs

We have previously shown that sEVs were enriched in fractions 2 and 3 (F2 and F3) when the TSU method is employed for sEVs isolation. In addition, we demonstrated that apolipoprotein contamination was lower in F2 and F3 compared to other fractions, which qualified these fractions to perform EV‐based functional studies (Chetty et al., 2022). The steps involved in sEVs isolation using TSU are illustrated in Figure 1a. As previously shown, MV4‐11 and K562 sEVs (F2 and F3) were characterized according to MISEV2018 guidelines. Negative staining of sEVs with phosphotungstic acid and subsequent observation in transmission electron microscopy (TEM) demonstrated that the particles present in F2 and F3 fractions possessed intact membrane sEVs morphology (Figure 1b). Particle size distribution of MV4‐11 and K562 sEV fractions (F2 and F3) was determined using Nanoparticle Tracking Analyzer (NTA) (Figure 1c). NTA data showed that most of the particles in MV4‐11 and K562 sEV fractions range from 70 to 200 nm. Mean values of particle number and free protein concentration determined using NTA and micro‐BCA assay, respectively, were depicted in Figure 1d. Furthermore, western blot analysis demonstrated that sEVs were enriched with classical EV markers such as syntenin, TSG101, and Hsp70 in fractions F2 and F3 without cellular debris contamination (calnexin) (Figure 1e).

### 3.2 MV4‐11 and K562 sEVs influence the function of BM‐MSCs

**FIGURE 2:**
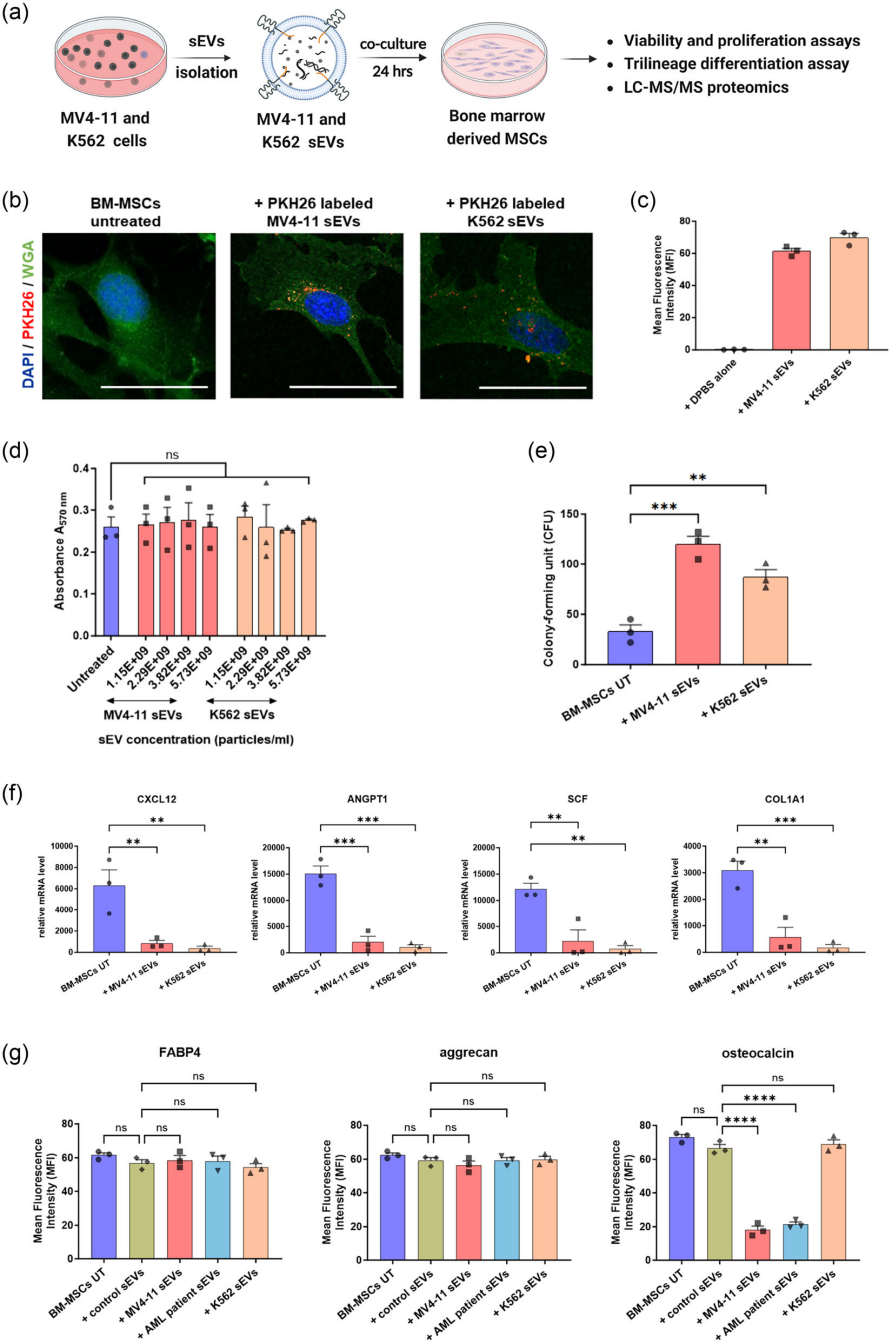
MV4‐11 and K562 sEVs influence BM‐MSCs differentiation. (a) Schematic workflow of bone marrow‐derived mesenchymal stem cells (BM‐MSCs) treatment with MV4‐11 and K562 sEVs. Small EVs were incubated with BM‐MSCs with a ratio of 50:1 (sEV particle number/recipient cells). Analysis of PKH26‐labelled MV4‐11 and K562 sEVs transferred into BM‐MSCs after 24 h using (b) confocal microscopy and (c) flow cytometry. Representative confocal images showing cell nuclei (DAPI, blue), cell membrane (WGA‐Alexa488, green) and PKH26‐labelled leukaemia sEVs (PKH26, red). Scale bars‐ 10 µm. (d) MTT assay of BM‐MSCs treated with different MV4‐11 and K562 sEVs concentrations for 24 h. (e) Colony‐forming unit (CFU) assay performed on BM‐MSCs treated with leukaemia sEVs for three weeks. (f) Relative mRNA expression of various genes important for normal hematopoietic function in bone marrow microenvironment. (g) Trilineage differentiation of BM‐MSCs treated with leukaemia and healthy control sEVs. Graphs showing the influence of adipogenic (FABP4), chondrogenic (aggrecan) and osteogenic (osteocalcin) differentiation of BM‐MSCs due to the sEVs treatment. Data shown in c‐g are mean ± S.E.M. obtained from three independent experiments, and statistical significance is calculated in reference to either untreated BM‐MSCs or BM‐MSCs treated with healthy control sEVs (****p < 0.0001, ***p < 0.001, **p < 0.01, and ns‐ non‐significant).

To understand how proteins associated with leukaemia sEVs influenced the function of BM‐MSCs, we incubated BM‐MSCs with sEVs derived from leukaemia cells. When TSU method is employed for sEVs isolation, it is already known that we obtain majority of sEVs in fractions F2 and F3, therefore we mixed these two fractions to treat with BM‐MSCs. The schematic workflow involved is shown in Figure 2a. To confirm the aspect that leukaemia sEVs internalize into BM‐MSCs to exhibit their biological function, MV4‐11 and K562 sEVs were labelled with lipophilic red fluorescent membrane dye (PKH26). Then, PKH26‐labelled MV4‐11 and K562 sEVs were incubated with BM‐MSCs with a ratio of 50:1 (sEV particle number/recipient cells) for 24 h and subsequently analysed by confocal microscopy and flow cytometry. Both analyses showed that a major proportion of PKH26‐labeled leukaemia sEVs were uptaken and internalized into BM‐MSCs (Figure 2b,c). Next, MTT assay performed on BM‐MSCs treated with increasing concentrations of leukaemia sEVs (1.15 × 10^9^ – 5.73 × 10^9^) demonstrated that leukaemia sEVs do not influence the viability of BM‐MSCs (Figure 2d). In addition, apoptosis assay performed on these cells confirmed that BM‐MSCs were not undergoing apoptosis or necrosis due to the leukaemia sEVs treatment (Figure S2a). On the other hand, colony‐forming unit (CFU) assay showed that MV4‐11 and K562 sEVs increased the proliferation potential of BM‐MSCs (Figure 2e).

To determine if leukaemia sEVs affect the haematopoiesis‐supporting function of BM‐MSCs, cells treated with leukaemia sEVs were screened for CXCL12, ANGPT1, COL1A1 and SCF gene expression. These proteins are secreted by BM‐MSCs that play a key role in maintaining normal haematopoiesis in the BMM. In concordance with the previous publications (Ghanam et al., 2023; Jafarzadeh et al., 2019; Kumar et al., 2018), we observed that MV4‐11 and K562 sEVs significantly downregulated the expression of these key genes in BM‐MSCs (Figure 2f). Next, MSC trilineage differentiation in vitro assays were performed on these BM‐MSCs treated cells to evaluate if leukaemia sEVs have any functional role in influencing the differentiation of BM‐MSCs. Adipogenesis, chondrogenesis, and osteogenesis differentiation of BM‐MSCs treated with leukaemia sEVs were determined using FABP4, aggrecan and osteocalcin, respectively. Indeed, we found that both MV4‐11 and K562 sEVs do not influence the adipogenic and chondrogenic differentiation potential of BM‐MSCs, whereas MV4‐11 sEVs decreased the osteogenic differentiation of BM‐MSCs to osteoblasts significantly (Figure 2g).

### 3.3 Identification of sEV protein cargo that influences the osteogenic differentiation of BM‐MSCs

**FIGURE 3:**
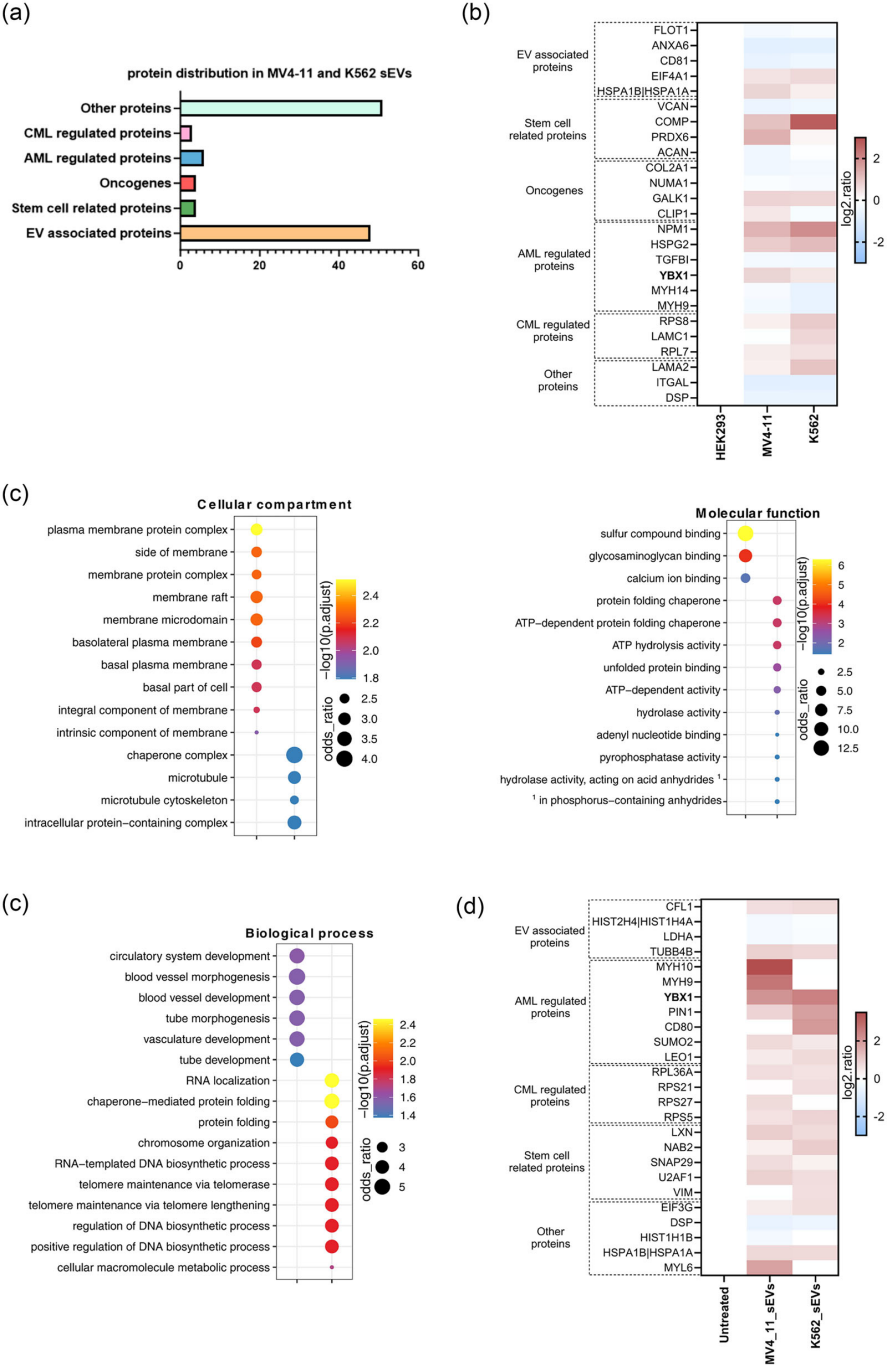
Identification of AML‐derived sEV protein targets that influence osteogenic differentiation of BM‐MSCs. (a) Different classes of proteins enriched in both MV4‐11 and K562 sEVs (CML‐chronic myeloid leukemia). (b) Heat map showing the different classes of proteins contained in leukaemia sEVs. (c) Gene ontology (GO) enrichment analysis of the proteome of MV4‐11 and K562 sEVs. BM‐MSCs were treated with leukaemia sEVs with a ratio of 50:1 (50 sEV particles per cell). (d) Heat map illustrating the different classes of proteins contained in BM‐MSCs treated with leukaemia sEVs.

To identify the sEV protein target derived from MV4‐11 sEVs responsible for the disturbance of osteogenic differentiation of BM‐MSCs, TMT multiplexed LC‐MS/MS proteomics was performed on both leukaemia sEVs alone and BM‐MSCs treated with leukaemia sEVs. BM‐MSCs were treated with leukaemia sEVs in the ratio of 50:1 (sEV particle number/recipient cells). BM‐MSCs incubated with healthy or diseased plasma sEVs were not included in this experiment since human plasma sEVs are very heterogeneous, which could mislead the targets. In this regard, in line with other studies (Cochran & Kornbluth, 2021; Sanwlani et al., 2023), for leukaemia sEV protein cargo analysis, we used HEK293 sEVs as a control for the data normalization. The rationale behind using HEK293T sEVs for the normalization is that it helps to neglect the protein cargoes that are common in any sEVs and to determine the protein cargoes that are specific for AML or CML.Please refer to the supporting information section for the complete proteomics data set. A total of 116 quantitative proteins were found to be common in both MV4‐11 and K562 sEVs. These proteins were then matched with the list of the proteins indexed in MISEV2018 guidelines (Thery et al., 2018) and in EV molecular databases such as Vesiclepedia (Kalra et al., 2012) and Exocarta (Keerthikumar et al., 2016). The distribution of different classes of proteins identified in both MV4‐11 and K562 sEVs is illustrated in Figure 3a and the complete list is shown in Supplementary Excel Sheet 2. We elucidated that 48 proteins out of 116 proteins found in both MV4‐11 and K562 sEVs are the proteins that are associated with EV secretion or biogenesis. In addition, we also found some leukaemia‐specific proteins, oncogenes (COL2A1, CLIP1, NUMA1 and GALK1), and stem cell‐regulated proteins (ACAN, VCAN, COMP and PRDX6). Interestingly, leukaemia regulated proteins such as NPM1, HSPG2, RPL7 and YBX1 were higher in MV4‐11 and K562 sEVs compared to HEK293 sEVs (Figure 3b).

Next, gene ontology (GO) enrichment analysis revealed that proteins associated with different biological processes (BP), cellular compartments (CC) and molecular functions (MF) are contained in MV4‐11 and K562 sEVs (Figure 3c). Especially the proteins involved in different BP, such as RNA localization, protein folding and DNA biosynthesis were enriched more in MV4‐11 sEVs. Furthermore, proteins associated with different MF, including sulfur compound binding, and glycosaminoglycan binding, are contained more in MV4‐11 sEVs.

On the other hand, LC‐MS/MS quantitative proteomics performed on BM‐MSCs treated with leukaemia sEVs revealed that the proteins associated with various pre‐ and post‐transcriptional events are particularly elevated on BM‐MSCs treated with leukaemia sEVs (Figure S2b). Importantly, proteins that have a regulatory role in mRNA metabolism and gene expression are influenced in BM‐MSCs due to the treatment of leukaemia sEVs. Especially, RNA‐binding protein, such as YBX1 that is necessary for tumour progression and metastasis (Evdokimova et al., 2009; Kwon et al., 2018), including the survival of myeloid leukaemia cells (Feng et al., 2021), is considerably upregulated on BM‐MSCs treated with MV4‐11 sEVs. Additionally, eukaryotic translation initiation factor 3 subunit G (Eif3g) that has an essential role in the Wnt signalling pathway and myosin heavy chain 10 (MYH10) that acts as a biomarker in platelet disorders (Antony‐Debre et al., 2012; Bluteau et al., 2012) are upregulated on BM‐MSCs treated with MV4‐11 sEVs (Figure 3d).

Analysing the proteomics data of MV4‐11 sEVs and BM‐MSCs treated with MV4‐11 sEVs together showed that YBX1, which plays a key role in AML development and maintenance is significantly upregulated in both the samples. Consistent with our observation, Perner et al. demonstrated that YBX1 is particularly upregulated in bone marrow biopsies of AML patients compared to healthy donors and the increased YBX1 expression is associated with AML progression (Perner et al., 2022). On the other hand, our proteomics data revealed that YBX1 is also upregulated in small EVs derived from AML (MV4‐11) cells. There is a high possibility that YBX1 derived from MV4‐11 sEVs could be the potential factor for the disturbance of osteogenic differentiation of BM‐MSCs.

### 3.4 Y‐box binding protein 1 (YBX1) in AML‐derived sEVs

**FIGURE 4:**
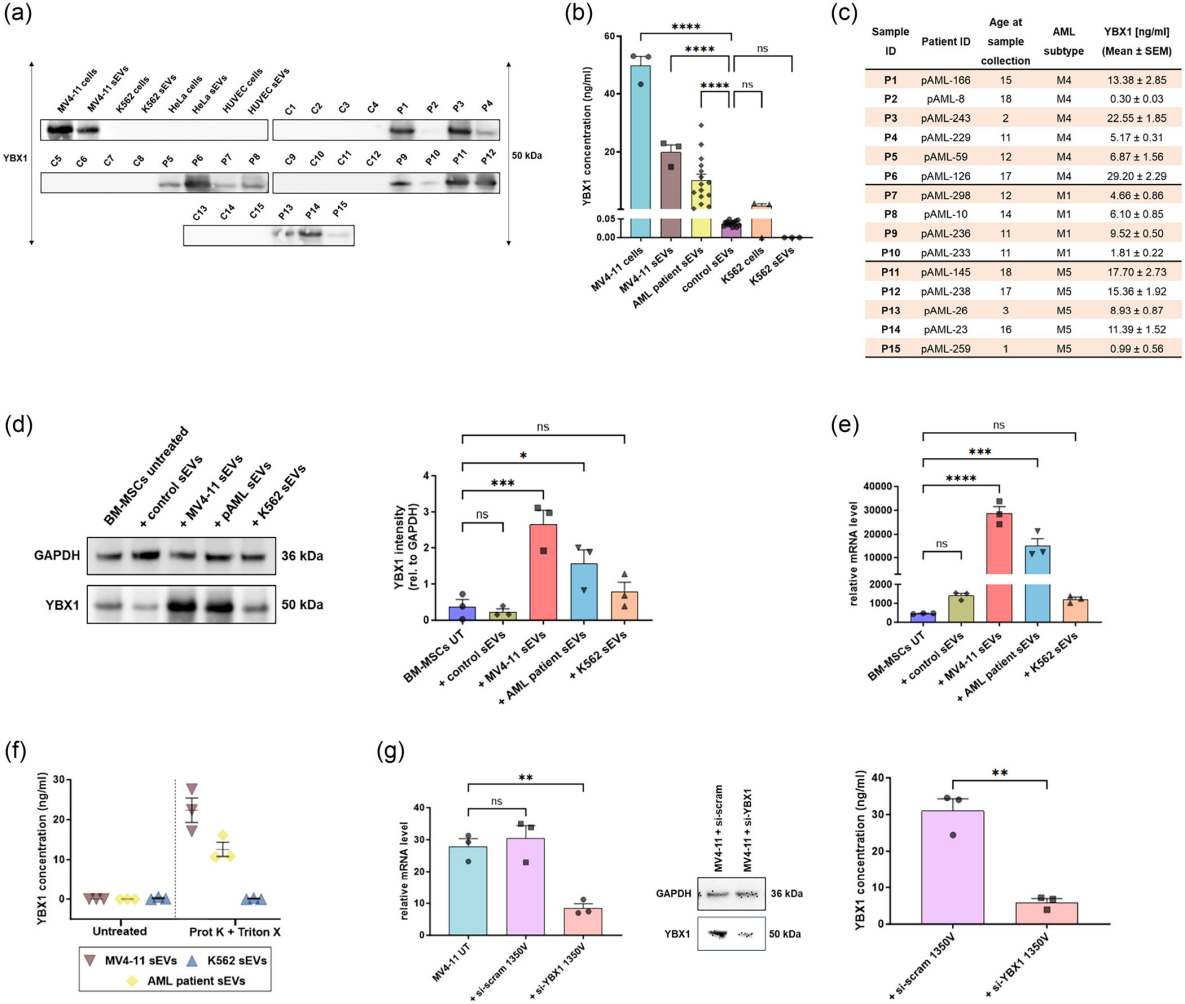
Upregulation of YBX1 protein cargo inside AML‐derived sEVs. (a) Western blot analysis of YBX1 in various AML and non‐AML samples. C1‐C15 and P1‐P15 indicates sEVs isolated from peripheral blood plasma of 15 paediatric healthy controls and 15 paediatric AML patients, respectively. (b) Quantification of YBX1 expression using ELISA (*N* = 15 for paediatric healthy control and AML patient sEVs). (c) Table showing the level of YBX1 (ng/mL) on various paediatric AML patients sEVs. Evaluation of YBX1 expression on BM‐MSCs treated with AML and non‐AML sEVs. sEVs were incubated with BM‐MSCs in the ratio of 50:1 (50 sEV particles per cell). (d) Immunoblotting of GAPDH and YBX1 on untreated BM‐MSCs and BM‐MSCs treated with different sEVs. Quantification of YBX1 relative to corresponding GAPDH signal. (e) Relative YBX1‐mRNA level on untreated BM‐MSCs and BM‐MSCs treated with different sEVs using qRT‐PCR. (f) Demonstration of the location of YBX1 cargo in leukaemia sEVs by incubation with Proteinase K (Prot K) and Triton X. Electroporation‐based downregulation of YBX1 in MV4‐11 cells. (g) Determination of YBX1 relative mRNA and protein level on MV4‐11 cells transfected with si‐YBX1 using qRT‐PCR, ELISA and western blot. Data illustrated in b, e, f and g for the cell line and their corresponding sEVs are mean ± S.E.M. of *n* = 3 experiments. All *p* values were calculated using one‐way ANOVA with Dunnett's multiple comparisons test except the ELISA analysis in f in which *p*‐value was calculated using an unpaired *t*‐test (****p < 0.0001, ***p < 0.001, **p < 0.01, *p < 0.05 and ns‐ non‐significant).

To affirm the fact that YBX1 is upregulated in AML‐sEVs, western blot for YBX1 was performed with various AML (MV4‐11 cells and their sEVs, sEVs from blood plasma of fifteen paediatric AML patients) and non‐AML samples (K562, HeLa, HUVEC cells and their sEVs, sEVs isolated from blood plasma of fifteen paediatric healthy controls) (Figure 4a). Then, YBX1 expression level was quantified using ELISA. Indeed, we found that YBX1 was overexpressed in MV4‐11 cells and their corresponding sEVs compared to non‐AML cells (Figure 4b and Figure S2c). Furthermore, compared with healthy control sEVs, we determined that YBX1 was upregulated on most paediatric AML patient sEVs. However, the level of YBX1 varies in each paediatric AML patient sEVs and is not correlated with AML subtype (M1, M4 and M5) (Figure 4c). Next, YBX1 expression was checked on BM‐MSCs treated with various AML sEVs (MV4‐11 and paediatric AML patient) and non‐AML sEVs (paediatric healthy control and K562). sEVs were added to BM‐MSCs with a ratio of 50:1 (50 sEV particles per cell). In line with the previous result, we also uncovered that YBX1 expression was significantly increased on BM‐MSCs treated with MV4‐11 and paediatric AML patient sEVs (Figure 4d,e), compared to healthy control sEVs.

Next, leukaemia sEVs were incubated with Proteinase K and subsequently lysed with Triton X following protease inhibitor treatment to reveal whether YBX1 is associated with the outer EV membrane or located inside the vesicles. Our results confirmed that the YBX1 protein associated with leukaemia sEVs is indeed located inside the vesicles (Figure 4f). YBX1 was then downregulated on MV4‐11 cells using electroporation‐based siRNA transfection against YBX1‐mRNA. Electroporation conditions such as 1350 volt, 35 ms, and 1 pulse were found to be suitable to downregulate YBX1 expression in MV4‐11 cells without affecting their cell viability (Figure S2d,e). The extent of YBX1 downregulation in MV4‐11 cells was further confirmed using ELISA and qRT‐PCR (Figure 4g). Next, sEVs were isolated from MV4‐11 cells in which YBX1 was downregulated, and afterward, the concentration of YBX1 was assessed using ELISA. We found that YBX1 concentration from MV4‐11 si‐YBX1 sEVs was remarkably less compared to MV4‐11 si‐scramble (Figure S2f).

### 3.5 YBX1 containing AML‐sEVs influence the osteoblastic differentiation of BM‐MSCs

**FIGURE 5:**
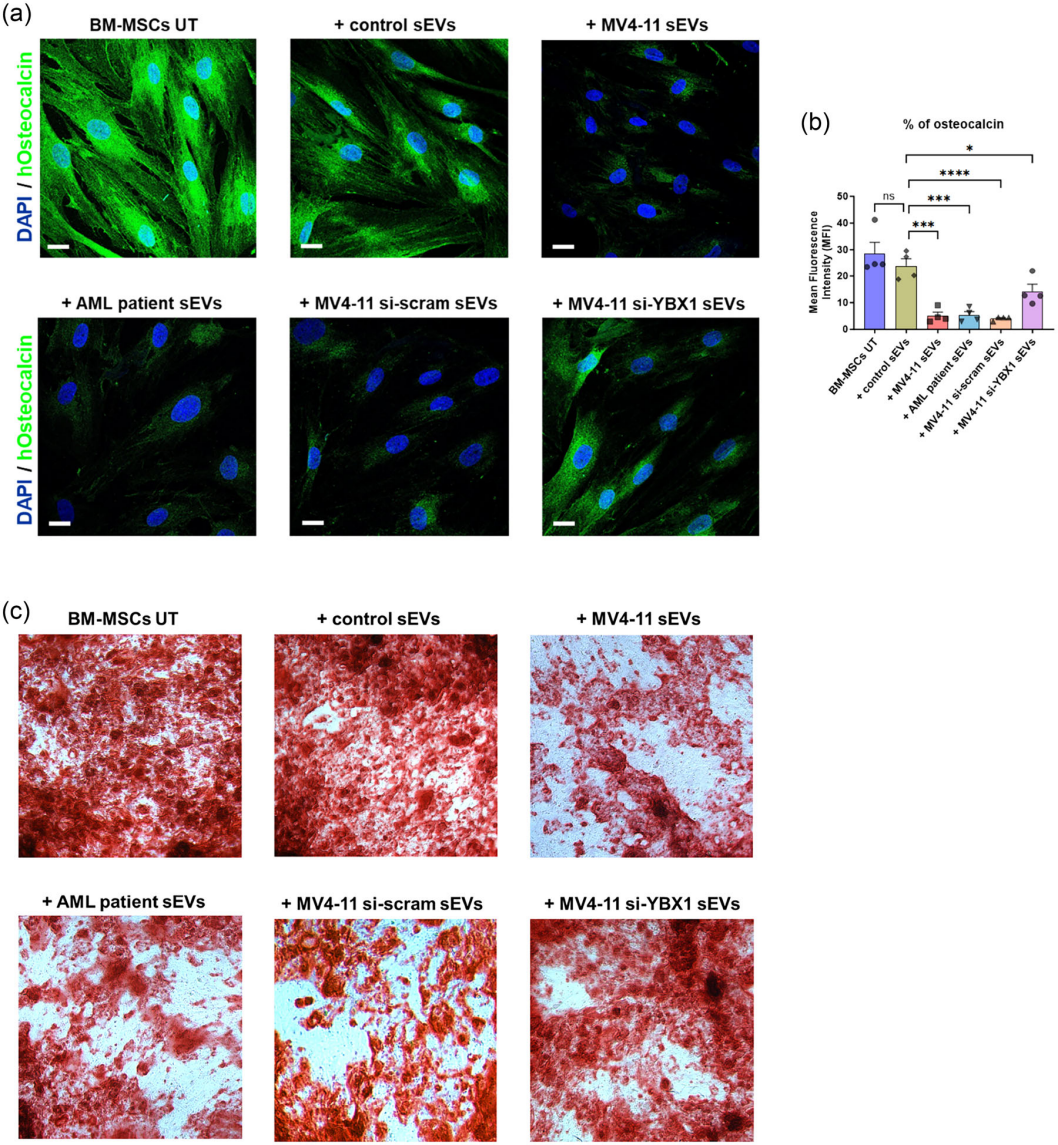
Osteogenic differentiation analysis of BM‐MSCs. Small EVs were incubated with BM‐MSCs in the ratio of 50:1 (50 sEV particles per cell). (a) Representative confocal images showing the osteoblastic differentiation of BM‐MSCs treated with AML and non‐AML sEVs. Cell nuclei are shown in blue (DAPI), and the osteogenesis marker (osteocalcin) is shown in green. Scale bars—10 µm. (b) Mean Fluorescence Intensity (MFI) of osteocalcin in BM‐MSCs treated with different sEVs expressing osteocalcin. For each condition, MFI average was calculated from at least four confocal images. *p* values were calculated using one‐way ANOVA with Dunnett's multiple comparisons (****p < 0.0001, ***p < 0.001, *p < 0.05 and ns‐ non‐significant). (c) Alizarin red staining of BM‐MSCs treated with AML and non‐AML sEVs.

At day 0, BM‐MSCs were treated with various sEVs including MV4‐11 si‐scramble sEVs and MV4‐11 si‐YBX1 sEVs with a ratio of 50:1 (50 sEV particles per cell). After 21 days, confocal microscopy‐based osteoblastic differentiation analysis using osteocalcin was performed. Confocal imaging precisely illustrated considerable reestablishment of osteoblastic differentiation of BM‐MSCs, when MV4‐11 si‐YBX1 sEVs were added compared to MV4‐11 si‐scramble sEVs (Figure 5a,b). To further confirm the involvement of YBX1 in the osteoblastic differentiation of BM‐MSCs, the same analysis was performed by overexpressing YBX1 on BM‐MSCs using pDESTmycYBX1 (Figure S3a,b). In comparison with the transfection control and empty vector, we found that osteoblastic differentiation was decreased to great extent on BM‐MSCs due to YBX1 overexpression.

Additionally, alizarin red staining was performed to stain the calcium‐containing osteocytes in differentiated BM‐MSCs. In line with the microscopy data, we found substantial improvement in the osteogenic differentiation of BM‐MSCs when cultured with MV4‐11 si‐YBX1 sEVs (Figure 5c). To reveal other sEV protein cargoes that cooperate along with YBX1 to regulate BM‐MSCs osteogenic differentiation, we characterized the MV4‐11 si‐YBX1 sEVs by LC‐MS/MS proteomics (Figure S3c). In general, we found that the expression of some sEV protein cargoes was affected in MV4‐11 sEVs due to YBX1 downregulation. Interaction of these sEV protein cargoes with YBX1 protein cargo to decrease osteoblastic differentiation will be addressed in our future research. Altogether, these results demonstrate that YBX1 containing small EVs derived from AML is one of the key players that reduce the BM‐MSCs differentiation to osteoblasts.

## 4 DISCUSSION

In acute myeloid leukaemia (AML), it is well known that abnormal secretion of pro‐inflammatory cytokines remodels the bone marrow microenvironment (BMM) in favour of AML blast survival, AML progression, and treatment resistance (Kupsa et al., 2012; Luciano et al., 2022). However, few recent studies have demonstrated that small extracellular vesicles (sEVs) released by AML cells transform the BMM into a leukaemia‐permissive microenvironment. These studies showed that AML‐sEVs influence normal haematopoiesis in BMM by acting as an essential communicator for the reciprocal interaction between BM‐MSCs and AML blasts, leading to poor prognosis and chemotherapy resistance (Chen et al., 2019; Huan et al., 2013; Kumar et al., 2018; Zhang et al., 2022). However, none of these studies clearly demonstrated which sEV cargo derived from AML influences the function of BM‐MSCs in BMM.

In line with our previous publication (Ghanam et al., 2023), we found that leukaemia‐derived sEVs increased the proliferation of BM‐MSCs without influencing the cell viability. In addition, Huan et al. showed that primary AML‐derived exosomes influenced the haematopoietic function of BM‐MSCs by negatively modulating the transcription factors involved in haematopoiesis control (Huan et al., 2013). In the same way, we observed that leukaemia‐derived sEVs downregulated the expression of various genes (CXCL12, ANGPT1, COL1A1 and SCF) in BM‐MSCs, vital to maintaining normal haematopoiesis in BMM.

On the other side, Kumar et al. demonstrated that BM‐MSCs treated with AML‐derived exosomes resulted in increased adipogenic and decreased osteogenic and chondrogenic differentiation due to the overexpression of osteoblast inhibitor, DKK1 in stromal cells (Kumar et al., 2018). In addition, Zhang et al. illustrated to some extent that AML cells co‐cultured with BM‐MSCs resulted in increased adipogenesis ability and decreased osteoblastic differentiation capacity (Zhang et al., 2022). In contrast to these studies, we found that BM‐MSCs treated with AML‐sEVs decreased only osteogenic differentiation but not adipogenic or chondrogenic differentiation.

Osteogenic differentiation is essential to support haematopoiesis in the BMM since osteoblasts are necessary for the normal functioning of hematopoietic stem cells (HSCs). Many pre‐clinical and clinical studies previously reported that BM‐MSCs derived from AML patients have reduced osteogenic differentiation potential compared with healthy donors (Azevedo et al., 2022), especially in high‐risk AML patients (Diaz de la Guardia et al., 2017). Notably, high osteocalcin level in the peripheral blood is positively correlated with disease status (Geyh et al., 2016) and overall survival (Chen et al., 2020). Adding an important point to these studies, we evidenced through in vitro trilineage differentiation assay that AML‐sEVs reduce the osteoblastic differentiation of BM‐MSCs, and the observed effect is likely due to the RNA‐binding protein associated with AML‐sEVs, YBX1. Nevertheless, this observation needs to be further validated in in vivo models.

As a key regulator of transcription and translation, RNA‐binding proteins are often dysregulated in various cancers (Pereira et al., 2017). Increasing evidence suggests that YBX1 plays a crucial role in AML development and maintenance (Feng et al., 2021; Perner et al., 2022; Zhou et al., 2021). These studies demonstrated that YBX1 mediates the translation of various oncogenic transcripts, including MYC, which leads to increased proliferation of AML cells. However, they have not addressed the functional interaction of YBX1 with hematopoietic or non‐haematopoietic components of BMM. Furthermore, Feng et al. (2021) demonstrated that the expression of YBX1 is significantly upregulated in AML cells (Feng et al., 2021). In line with this data, we observed that YBX1 expression is enormously upregulated not only in AML cells but also in sEVs derived from most paediatric AML patients compared to healthy controls. In future studies, after analysing YBX1 expression in large AML‐sEVs patient cohorts belonging to different subtypes, YBX1 could potentially serve as a new clinical biomarker for AML management.

Last, we observed that the downregulation of YBX1 in MV4‐11 cells remarkably improved the osteoblastic differentiation of BM‐MSCs. In addition, overexpression of YBX1 in BM‐MSCs substantially reduced their osteoblastic differentiation. Together, these data indicate that the YBX1 protein is one of the key players that reduce the BM‐MSCs’ differentiation to osteoblasts. On the other hand, we cannot ascertain that YBX1 derived from AML‐sEVs is the only responsible factor for the osteoblast differentiation blockage of BM‐MSCs. Because it is known that BM‐MSCs itself release an enormous number of sEVs that interact with several target cells in the tumour microenvironment, including natural killer and haematopoietic stem cells, leading to the development of AML cells proliferation, invasion and chemoresistance (Abbasi et al., 2022; Lyu et al., 2021).

Furthermore, we found that some of the other sEV protein cargoes are influenced due to YBX1 downregulation in AML cells. It is more likely that these protein cargoes cooperate with YBX1 cargo to reduce the osteogenic differentiation of BM‐MSCs. Therefore, further research elucidating the interaction of AML‐sEV‐derived YBX1 with other protein cargoes would help to understand the exact mechanism involved in the blockage of BM‐MSCs differentiation leading to AML progression that result in bone marrow failure.

## 5 CONCLUSION

In summary, our results demonstrated that YBX1 containing small extracellular vesicles derived from AML decrease the osteoblastic differentiation of BM‐MSCs. In the future, revealing the functional interaction of YBX1 with other sEV protein cargoes is vital to further understanding the BM‐MSCs osteoblastic differentiation blockage in AML, thereby enabling us to develop novel therapeutics for paediatric AML.

## AUTHOR CONTRIBUTIONS

**Venkatesh Kumar Chetty**: Conceptualization; data curation; formal analysis; investigation; methodology; validation; visualization; project administration; writing—original draft; writing—review and editing. **Jamal Ghanam**: Data curation; investigation; visualization; writing—review and editing. **Kristína Lichá**: Data curation; investigation. **Alexandra Brenzel**: Data curation; investigation. **Dirk Reinhardt**: Resources; supervision. **Basant Kumar Thakur**: Conceptualization; funding acquisition; methodology; validation; project administration; resources; supervision; writing—review and editing.

## CONFLICT OF INTEREST STATEMENT

The authors report no conflict of interest.

## GEOLOCATION INFORMATION

University Hospital Essen, Essen, Germany.

GPS: 51.4362442, 6.9893013

## Supporting information

Supplementary Information

Supplementary Information

Supplementary Information

Supplementary Information

**Figure S1**. Plasmid maps.
**Figure S2**. Bone marrow‐derived mesenchymal stem cells (BM‐MSCs) treated with leukemia sEVs.
**Figure S3**. Involvement of YBX1 in BM‐MSCs osteoblastic differentiation.

## Data Availability

Data available on request from the corresponding authors.
