# Supplementary material for: Y‐box binding protein 1 in small extracellular vesicles reduces mesenchymal stem cell differentiation to osteoblasts—implications for acute myeloid leukaemia
Source: J Extracell Vesicles. 2024 Mar 18;13(3):e12417. doi: 10.1002/jev2.12417 (PMC10948369; doi:10.1002/jev2.12417)
Supplement: Supplementary file 5 — Figure S1. Plasmid maps. Figure S2. Bone marrow‐derived mesenchymal stem cells (BM‐MSCs) treated with leukemia sEVs. Figure S3. Involvement of YBX1 in BM‐MSCs osteoblastic differentiation. [file JEV2-13-e12417-s004.docx]

Supplementary Figures

Y-box binding protein 1 in small extracellular vesicles reduces mesenchymal stem cell differentiation to osteoblasts - implications for acute myeloid leukemia

Venkatesh Kumar Chetty, Jamal Ghanam, Kristína Lichá, Alexandra Brenzel, Dirk Reinhardt and Basant Kumar Thakur


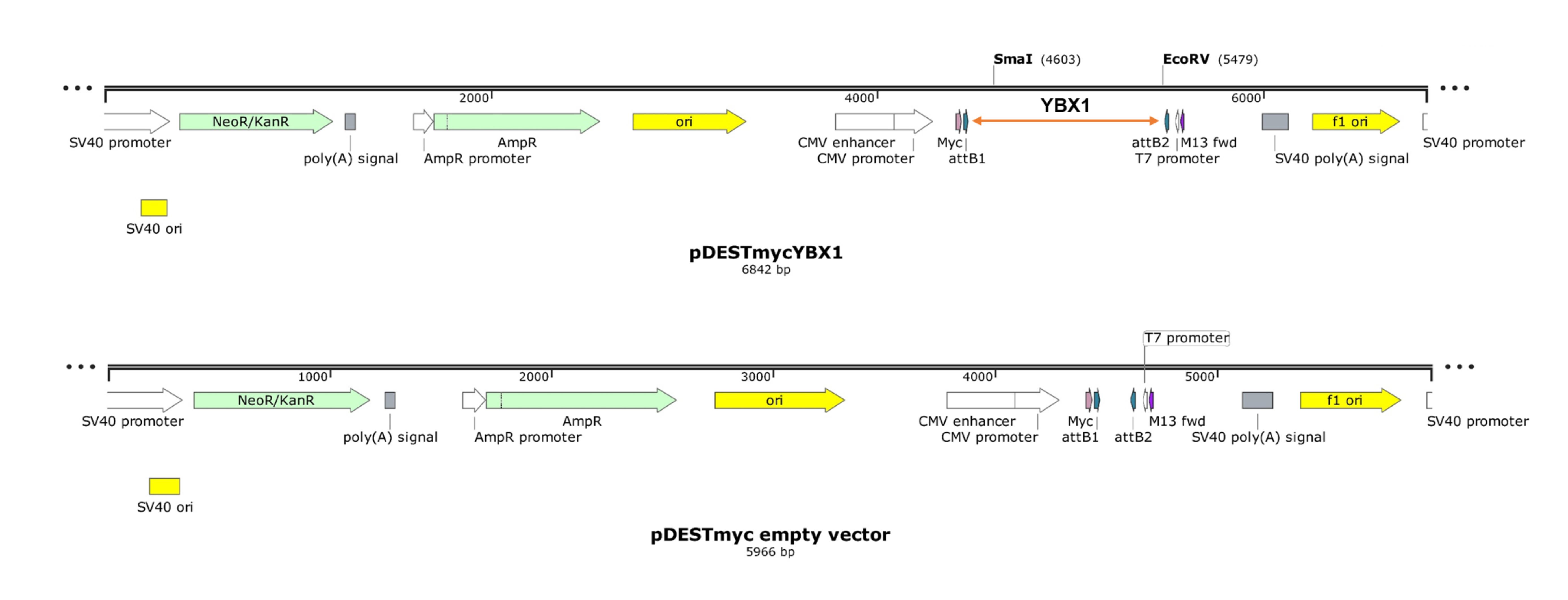


**Supplementary Figure S1. Plasmid maps.** Maps of pDESTmycYBX1 and pDESTmyc empty vector created with SnapGene® software (from Dotmatics; available at snapgene.com).


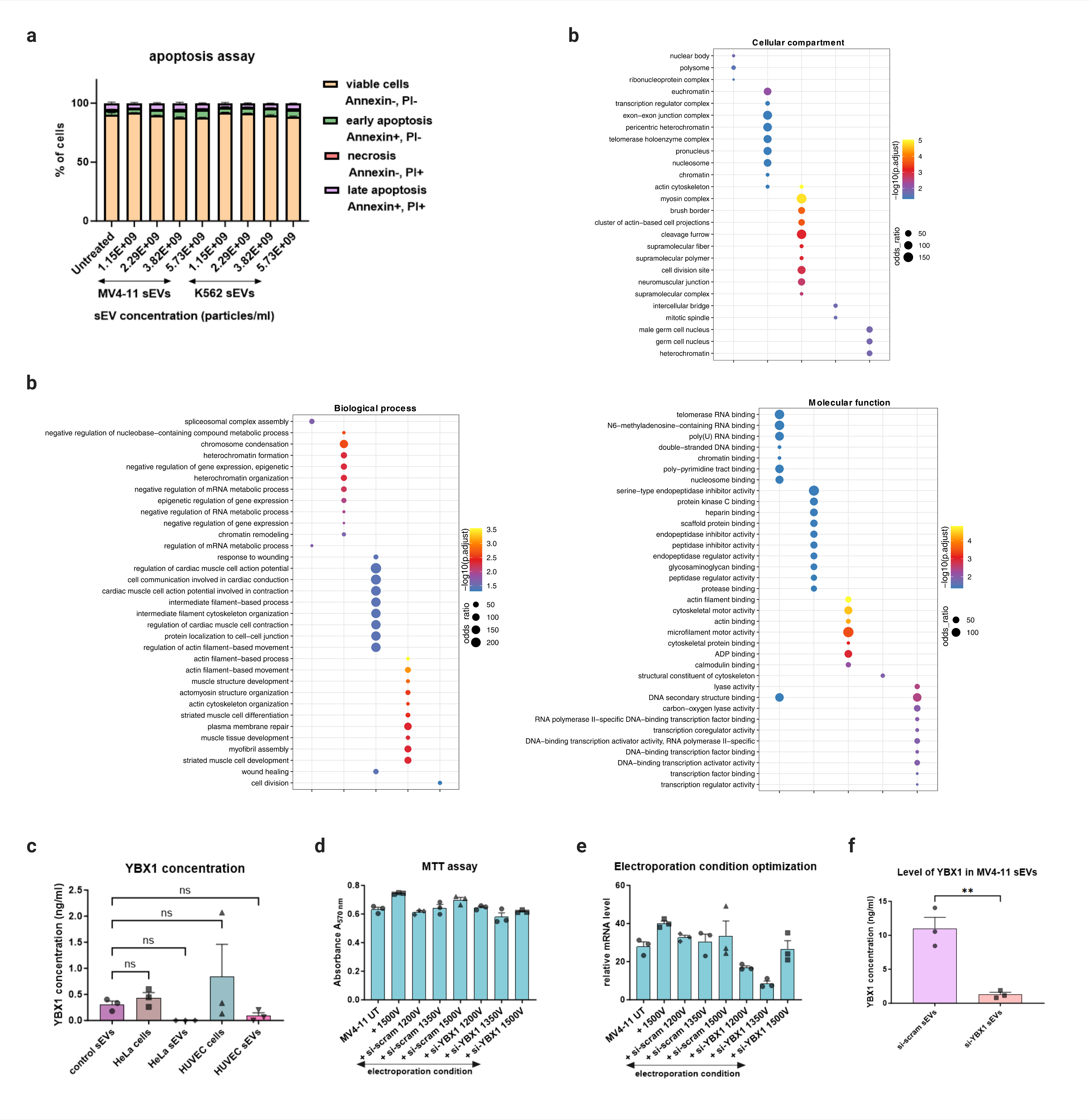


**Supplementary Figure S2. Bone marrow-derived mesenchymal stem cells (BM-MSCs) treated with leukemia sEVs.** (a) Cell apoptosis assay of BM-MSCs incubated with leukemia sEVs. (b) Gene ontology (GO) enrichment analysis of the proteome of BM-MSCs treated with MV4-11 and K562 sEVs. (c) Quantification of YBX1 protein in non-AML cells and their corresponding sEVs. (d) MTT assay of MV4-11 cells transfected with si-scramble and si-YBX1 under various electroporation conditions. (e) Relative mRNA level of YBX1 on MV4-11 cells transfected with si-scramble and si-YBX1 under different electroporation conditions. (f) Level of YBX1 on sEVs derived from MV4-11 si-scramble and MV4-11 si-YBX1 cells. Data illustrated in c-f are mean ± S.E.M obtained from three independent experiments, and statistical significance in c is calculated in reference to healthy control sEVs, and in f with reference to si-scramble (**p<0.01, and ns- non-significant).


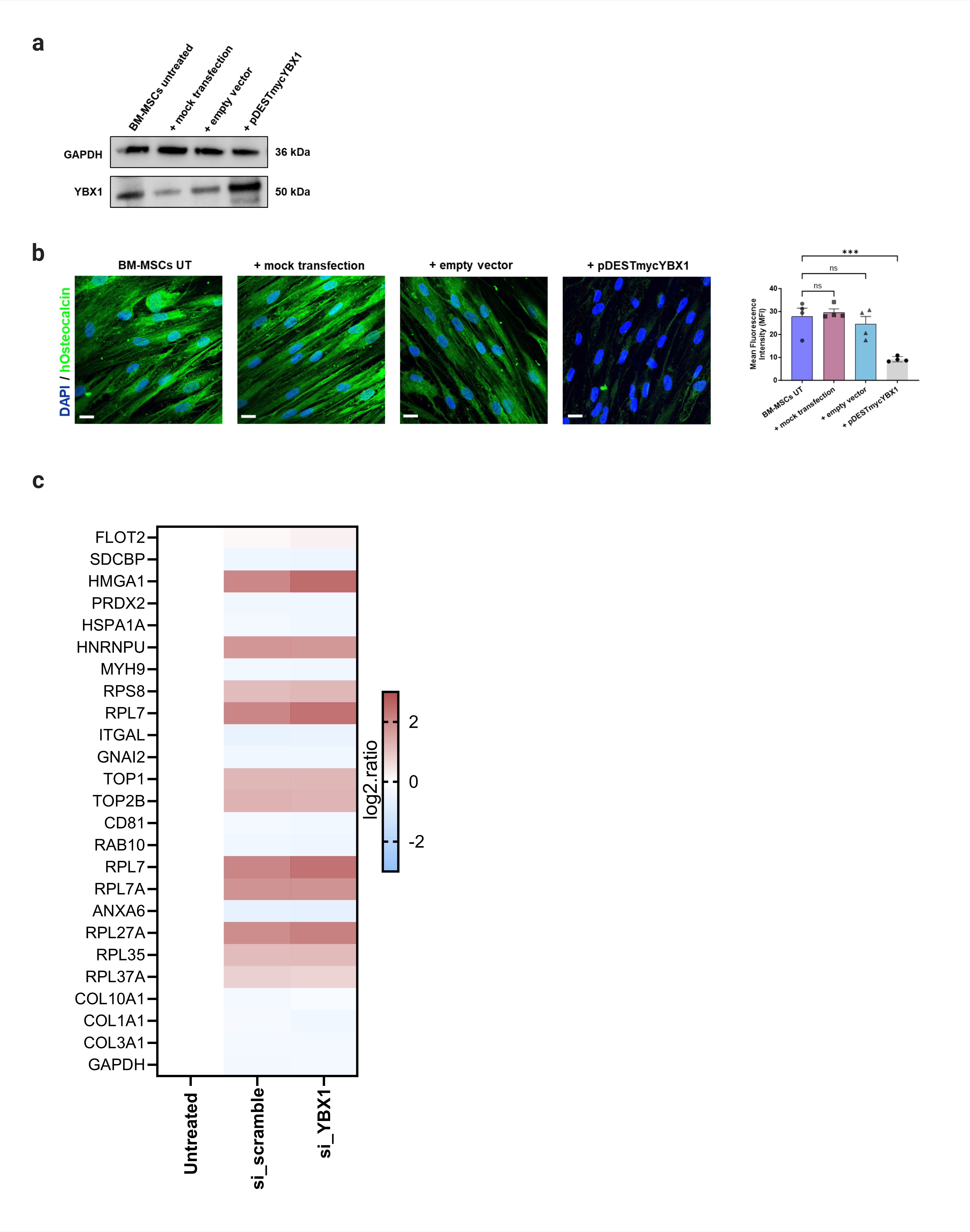


**Supplementary Figure S3. Involvement of YBX1 in BM-MSCs osteoblastic differentiation.** Overexpression of YBX1 in BM-MSCs using pDESTmycYBX1. (a) Western blot analysis of YBX1 on BM-MSCs transfected with pDESTmycYBX1 and empty vector. (b) Representative confocal images showing the osteoblastic differentiation of untreated BM-MSCs and BM-MSCs transfected with pDESTmycYBX1 and empty vector (cell nuclei- blue and osteocalcin- green). Scale bars - 10 µm. (c) Proteomics characterization of sEVs derived from MV4-11 si-YBX1 transfected cells. Heat map showing the expression of different sEV protein cargoes contained in untreated MV4-11 sEVs, MV4-11 si- scramble sEVs and MV4-11 si-YBX1 sEVs.
